# Interpretable multiparametric MRI radiomics-based machine learning model for preoperative differentiation between benign and malignant prostate masses: a diagnostic, multicenter study

**DOI:** 10.3389/fonc.2025.1541618

**Published:** 2025-05-05

**Authors:** Wenjun Zhou, Zhangcheng Liu, Jindong Zhang, Shuai Su, Yu Luo, Lincen Jiang, Kun Han, Guohua Huang, Jue Wang, Jianhua Lan, Delin Wang

**Affiliations:** ^1^ Department of Urology, The First Affiliated Hospital of Chongqing Medical University, Chongqing, China; ^2^ Department of Urology, Guang’an People’s Hospital, Guang'an, Sichuan, China; ^3^ Department of Urology, Neijiang Second People’s Hospital, Neijiang, Sichuan, China; ^4^ Department of Urology, Panzhihua Central Hospital, Panzhihua, Sichuan, China

**Keywords:** malignant prostate mass, multiparametric magnetic resonance imaging, radiomics, machine learning, interpretation

## Abstract

**Objective:**

The study aimed to develop and externally validate multiparametric MRI (mpMRI) radiomics-based interpretable machine learning (ML) model for preoperative differentiating between benign and malignant prostate masses.

**Methods:**

Patients who underwent mpMRI with suspected malignant prostate masses were retrospectively recruited from two independent hospitals between May 2016 and May 2023. The prostate mass regions in T2-weighted imaging (T2WI) and diffusion-weighted imaging (DWI) MRI images were segmented by ITK-SNAP. PyRadiomics was utilized to extract radiomic features. Inter- and intraobserver correlation analysis, t-test, Spearman correlation analysis, and the least absolute shrinkage and selection operator (LASSO) algorithm with a five-fold cross-validation were applied for feature selection. Five ML learning models were built using the chosen features. Model performance was evaluated with internal and external validation, using area under the curve (AUC), calibration curves, and decision curve analysis to select the optimal model. The interpretability of the most robust model was conducted via SHapley Additive exPlanation (SHAP).

**Results:**

A total of 567 patients were enrolled, consisting of the training (n = 352), internal test (n = 152), and external test (n = 63) sets. In total, 2,632 radiomic features were extracted from regions of interest (ROIs) of T2WI and DWI images, which were reduced to 18 via LASSO. Five ML models were established, among which the random forest (RF) model presented the best predictive ability, with AUCs of 0.929 (95% confidential interval [CI]: 0.885–0.963) and 0.852 (95% CI: 0.758–0.934) in the internal and external test sets, respectively. The calibration and decision curve analyses confirmed the excellent clinical usefulness of the RF model. Besides, the contributing relations of the radiomic features were uncovered using SHAP.

**Conclusions:**

Radiomic features from mpMRI combined with machine learning facilitate accurate preoperative evaluation of the malignancy in prostate masses. SHAP can disclose the underlying prediction process of the ML model, which may promote its clinical applications.

## Highlights

- Noninvasive mpMRI radiomics-based machine learning models were used to distinguish between benign and malignant prostate masses.
- The RF model demonstrated the highest predictive accuracy, with robust performance validated on external cohorts.
- SHAP analysis enhanced the interpretability of the RF model, facilitating clinical decision making in prostate cancer diagnosis.

## Introduction

Prostate cancer (PCa) is one of the most commonly diagnosed malignancies and a significant contributor to cancer-related mortality among men worldwide (1, 2). According to the World Health Organization, PCa represents approximately 15% of all new cancer cases in men, with substantial variation in incidence and mortality rates across regions (3, 4). Benign prostatic hyperplasia (BPH) is defined as a noncancerous enlargement of the prostate common in aging men (5–7). Accurate differentiation between PCa and BPH is crucial, as these conditions share overlapping symptoms, such as urinary difficulties, but differ vastly in prognosis and treatment requirements (8, 9). Misidentification between PCa and BPH can lead to under- or overtreatment, underscoring the need for precise diagnostic tools that can reliably distinguish between malignant and benign prostate conditions.

Traditional diagnostic tools for prostate conditions include prostate-specific antigen (PSA) testing, multiparametric MRI (mpMRI), digital rectal examination (DRE), and transrectal ultrasound (TRUS)-guided biopsy (10–14). Although PSA test has increased early detection, it lacks specificity, leading to unnecessary biopsies and potential overdiagnosis of low-risk tumors (12, 15). Although DRE and TRUS are helpful in diagnosing PCa, they involve invasive procedures that may bring about multiple complications. Imaging advancements, particularly the mpMRI, have increased diagnostic accuracy by enhancing lesion visualization and reducing reliance on invasive procedures (16–20). However, the evaluation of mpMRI images is highly dependent on the expertise of radiologists and can be subject to variability, highlighting the need for standardized and reproducible diagnostic tools. Therefore, there is a strong need for noninvasive, accurate diagnostic tools that can differentiate PCa from BPH while assessing tumor aggressiveness when malignancy is present.

Radiomics is an evolving field that converts medical images, such as MRI or CT, into quantitative data that can reveal underlying biological information about tumors (21, 22). It involves extracting features like texture, shape, and intensity, which may provide valuable insights into tissue composition and disease characteristics beyond what is visible in conventional imaging (23). MRI or CT radiomics-based machine learning (ML) models have shown potential in differentiating benign from malignant masses across various cancers, including lung, liver, and breast tumors (24–26). These findings underscore radiomics’ ability to improve diagnostic accuracy by capturing subtle variations in tissue that may not be visible to the naked eye. Several previous researchers have developed CT- or MRI-based radiomics models for differentiating malignant from benign prostate masses as well (27–29). However, existing research faces notable limitations. Most studies rely on relatively small, single-center cohorts, with few conducting external validations, limiting the models’ generalizability across broader clinical settings. Additionally, comparisons among different radiomics-based ML models are often lacking, and the interpretability of these models remains underexplored.

This study aims to develop and externally validate mpMRI radiomics-based ML models for preoperatively differentiating between malignant and benign prostate masses. The predictive performances of the established models are compared, and the most robust prediction model is interpreted using SHapley Additive exPlanations (SHAP).

## Methods

### Study cohorts

This retrospective, multicenter study involved two independent institutes: the First Affiliated Hospital of Chongqing Medical University (Center 1) and the Guang’an People’s Hospital (Center 2). The Institutional Review Board (IRB) of our hospital approved this study (approval number: K2023-599), and the patient’s informed consent requirement was waived. All study protocols were in accordance with the Declaration of Helsinki (30). The patients’ clinic-radiological features, MRI images, and whole-slide image were anonymized before all protocols.

Patients who underwent prostate biopsy or radical prostatectomy (RP) for pathological diagnosis between May 2016 and May 2023 were enrolled (Center 1, n = 813; Center 2, n = 157). RP pathology was used as the primary gold standard for cancer diagnosis, whereas for biopsy-only patients, a composite reference was established using multiparametric MRI and MRI/ultrasound fusion-targeted biopsy, combined with longitudinal follow-up. To minimize biopsy false negatives, a standardized biopsy protocol was employed, including MRI-targeted biopsy and centralized pathology review (31). Biopsy-negative patients with elevated PSA velocity underwent repeat biopsy or advanced biomarker testing. We excluded patients (1) without multiparametric MRI scans or with poor image quality (n = 170), (2) without complete clinic-pathological data (n = 87), (3) who received previous therapy or biopsy prior to MRI scans (n = 57), and (4) whose MRI images exhibited unrecognizable prostate mass boundaries (n = 77).

**Figure 1:**
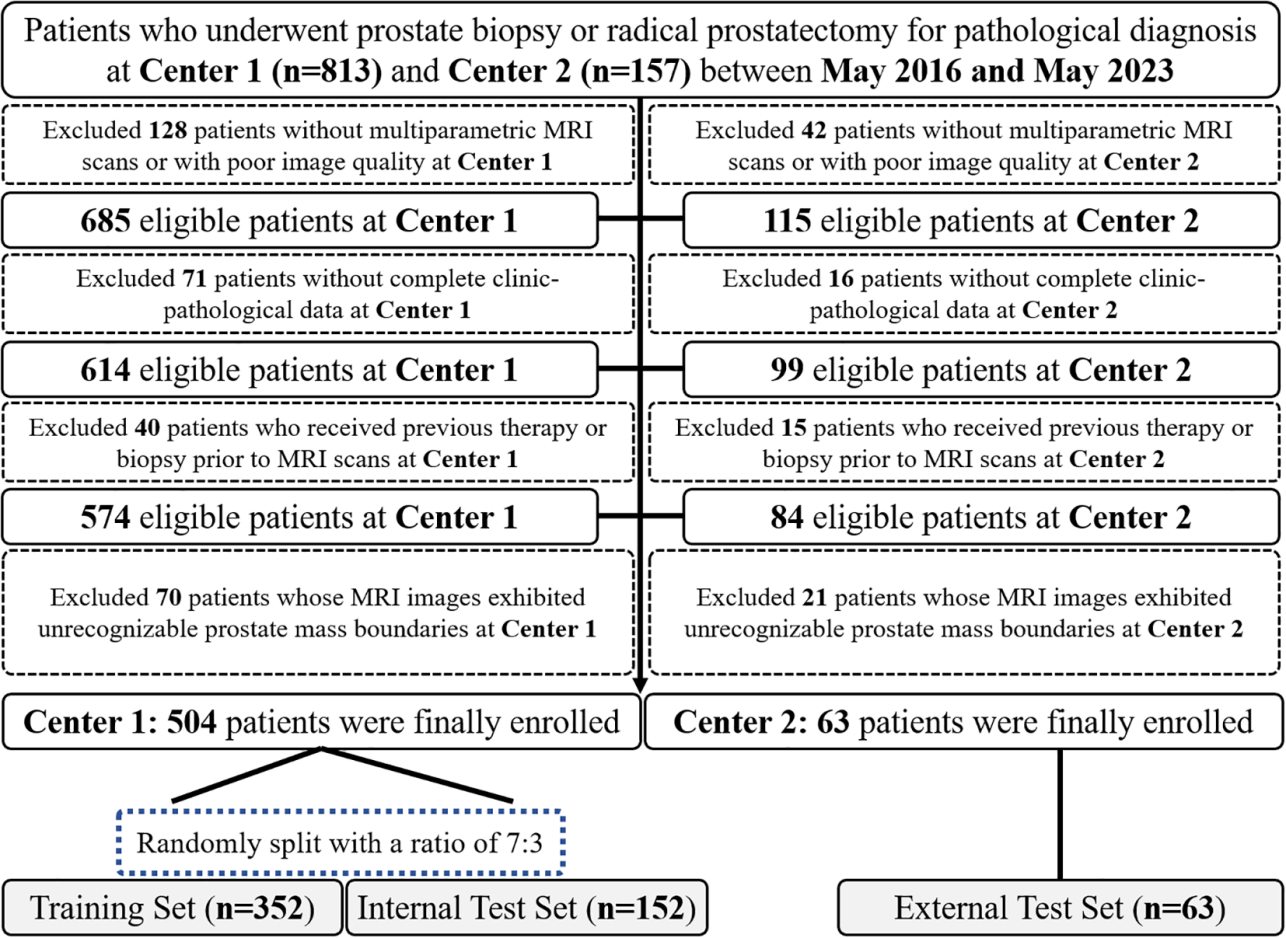
Patients’ recruitment flowchart of this multicenter study.

A total of 567 patients were finally recruited, consisting of 504 patients from Center 1 and 63 patients from Center 2. With a ratio of 7:3, patients from Center 1 were split into the training (n = 352) and internal test set (n = 152). Patients from Center 2 were assigned as the external test set (n = 63). The detailed patients’ recruitment flow is shown in **Figure 1**.

### Clinic-radiological features and histopathological evaluation

Clinical characteristics, including age, total prostate-specific antigen (tPSA), free prostate-specific antigen (fPSA), the ratio of fPSA to tPSA (fPSA/tPSA), and prostate-specific antigen density (PSAD), were collected via the electronic medical recording system. Radiological features such as prostate volume, seminal vesicle invasion (SVI), extracapsular extension (ECE), and lymph node invasion (LNI) were assessed by two experienced radiologists (both with over 8 years’ experience in urological image reading). The controversial cases were reevaluated by a third senior radiologist (with over 15 years’ experience in urological image reading).

The pathological data comprised the results of the transrectal ultrasound (TRUS) biopsy and the findings subsequent to radical prostatectomy. A systematic 12-core transrectal ultrasound (TRUS) biopsy was performed, with a minimum of two cores obtained from each target. In addition, needle biopsies were performed on the areas of the lesion identified on the MRI scans. The evaluation of the pathology slides was conducted by an experienced senior pathologist who was unaware of the MRI results and had accumulated over a decade of expertise in the analysis of prostate samples. Tumor classification was based on the 2016 WHO classification, with additional grading determined by the Gleason score (GS) and cancer group grades (32, 33).

### MRI examination and prostate mass region delineation

In this study, multiparametric MRI examinations were conducted on patients presenting with signs of prostate pathology. At Center 1, imaging was conducted using a high-resolution 3.0 T MR scanner (GE Discovery MR750W, General Healthcare, Milwaukee, USA) with an eight-channel abdominal surface coil. At Center 2, a 3.0 T MRI scanner (Philips Intera Achieva, Best, Netherlands) with a 32-channel body phased-array coil was used for image acquisition. T2-weighted imaging (T2WI) and diffusion-weighted imaging (DWI) served as the main sequences for subsequent feature extraction and analysis. T2WI was used to capture detailed anatomical structure, whereas DWI, alongside apparent diffusion coefficient (ADC) mapping, enabled quantitative assessment of tumor cellularity—a key indicator of malignancy. Detailed MRI parameters are provided in **Supplementary Figure S1**.

**Figure 2:**
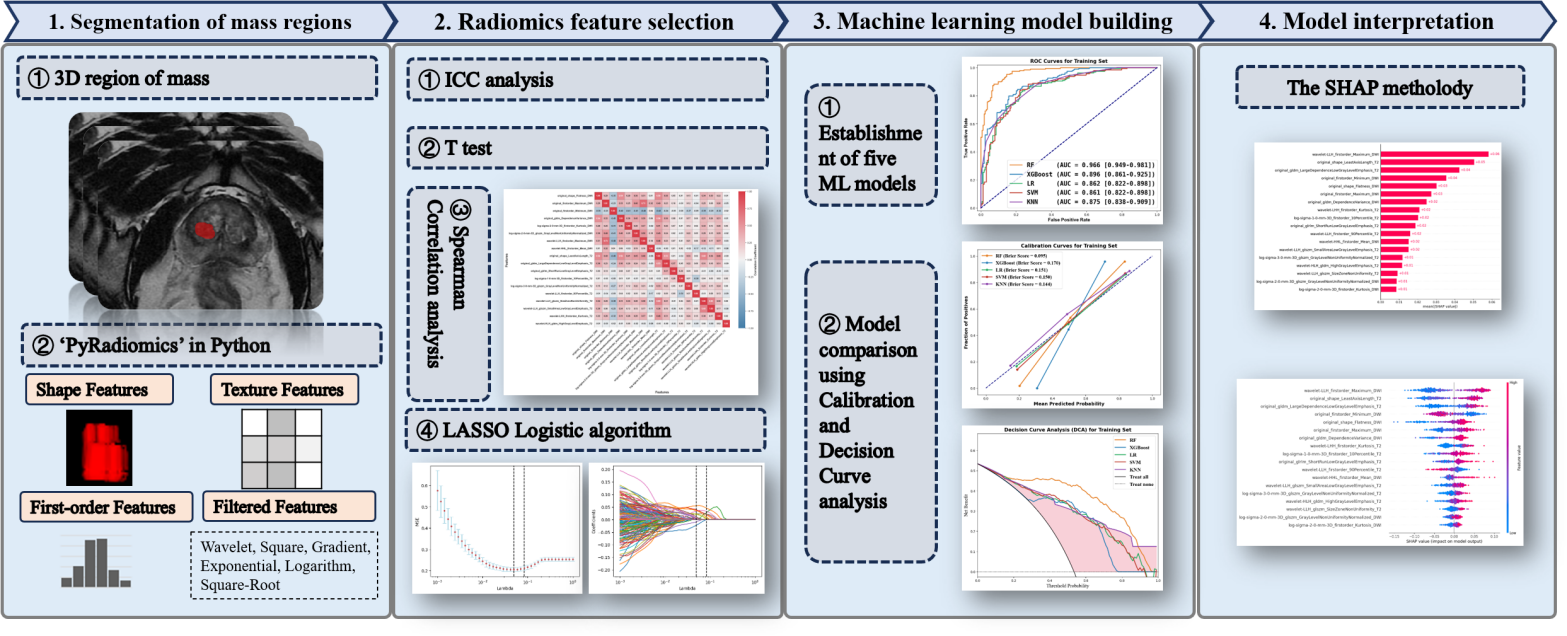
The overall workflow of this study.

Two independent radiologists (Readers A and B, both with over 8 years of experience in PCa diagnosis) who were blinded to the patients’ clinic-histopathological data delineated the prostate mass region, using the ITK-SNAP software (http://www.itksnap.org/pmwiki/pmwiki.php). Reader A firstly segmented the 3D region of interest (ROI) for all patients. Two weeks later, 50 patients were randomly selected and resegmented by Readers A and B for the calculations of inter- and intraobserver correlation coefficients (ICCs). The controversial cases were reevaluated by a third senior radiologist (Reader 3, with over 15 years of experience in PCa diagnosis). The Prostate Imaging–Reporting and Data System (PI-RADS) score was assessed when segmenting ROIs. The study workflow is illustrated in **Figure 2**.

### Radiomic feature extraction and selection

Prior to radiomic feature extraction, image preprocessing included normalization, resampling to consistent voxel spacing, and intensity standardization to ensure comparability across MRI scans. PyRadiomics in Python was utilized to extract radiomic features from 3D ROI of T2WI and DWI images. In each phase, 14 shape features, 18 first-order features, 75 texture features that derived from the original images, and 1,209 filtered features from the images after transformation (the image-transformation methods included exponential, gradient, logarithm, square, square-root, and wavelet) were extracted. The extracted radiomic features were standardized using Z-score normalization.

A four-step feature selection process was employed. First, inter- and intraobserver correlation analysis was conducted to calculate ICCs. Features with both inter- and intraobserver correlation coefficients more than 0.75 were considered highly reproduceable. Second, a t-test was employed to screen the significantly relevant features to malignant prostate mass. Third, a Spearman correlation analysis with a threshold of 0.80 was conducted to reduce redundant features. Lastly, the least absolute shrinkage and selection operator (LASSO) logistic algorithm with a five-fold cross-validation was employed to filter the optimal radiomic features subset for predicting malignant prostate mass.

### Machine learning model building and comparison

Five machine learning models, namely, random forest (RF), eXtreme Gradient Boosting (XGBoost), logistic regression (LR), support vector machine (SVM), and k-nearest neighbor (KNN), were employed to establish prognostic models for malignant prostate mass, using the selected radiomic features. Grid search with five-fold cross-validation was applied to optimize the hyperparameters for each classifier in the training set, which were further validated in the internal and external test sets (**Figure 2**). The receiver operating characteristic (ROC) curve analysis, area under the ROC curve (AUC), accuracy (ACC), sensitivity (SEN), specificity (SPE), positive predictive value (PPV), and negative predictive value (NPV) were calculated for the models’ performance evaluation. To compare the predictive performances and clinical usefulness of the constructed ML models, the DeLong test, calibration curve analysis with Brier score loss, and decision curve analysis were conducted. A lower Brier score indicated better model calibration.

### Interpretation of machine learning model

The most robust ML model was interpreted via the SHAP methodology, which is broadly applied in exploring the interpretability of ML models (34, 35). Based on the cooperative game theory, SHAP calculates each feature’s influence on model predictions by evaluating its marginal impact across all feature combinations, ensuring a balanced representation of feature importance. It offers interpretability on both a local scale by clarifying individual predictions and a global scale by summarizing the relative influence of features across the dataset.

### Statistical analysis

Statistical analysis was performed using SPSS 25.0 statistical software (SPSS, Armonk, NY, USA), R software (version 4.3.1; https://www.r-project.org/), and Python (version 3.8.0; https://www.python.org/). The Shapiro–Wilk test assessed normality for continuous variables, with normal data reported as mean ± SD and analyzed via t-tests; nonnormal data were given as medians with interquartile ranges (IQRs) and compared using Mann–Whitney U tests. Categorical data, shown as counts (percentages), were evaluated using chi-square or Fisher’s exact test. Based on the Youden index, optimal cutoff-based accuracy, sensitivity, specificity, PPV, and NPV were calculated, with 95% confidential intervals (CIs) estimated using 1,000 bootstraps. A significance threshold of P < 0.05 was applied throughout.

## Results

### Clinical characteristics

A total of 579 patients (mean age: 70.0 years, IQR: 65.0–75.0 years) were retrospectively enrolled from two centers. Of these, 249 cases (43.9%) were pathologically confirmed as benign prostate masses, and 318 cases (56.1%) were malignant. As shown in **Table 1**, there were no statistically significant differences among the training, internal test, and external test sets in terms of clinic-radiologic-histopathological characteristics, including age, tPSA, fPSA, fPSA/tPSA, PSAD, prostate volume, Gleason score, and the presence of SVI, LNI, and ECE, with all *P* values greater than 0.05.

**Table 1:** The clinical, radiological, and histopathological characteristics of the study cohorts.

Variable	All patients (n = 567)	Training set (n = 352)	Internal test set (n = 152)	External test set (n = 63)	*P* value
Pathological diagnosis, %					0.21
Benign	249 (43.9)	164 (46.6)	58 (38.2)	27 (42.9)	
Malignant	318 (56.1)	188 (53.4)	94 (61.8)	36 (57.1)	
Age, years	70.00 (65.00–75.00)	70.00 (65.00–75.00)	69.00 (65.00–76.00)	72.00 (67.00–77.00)	0.13
tPSA, ng/ml	17.35 (10.21–55.67)	17.51 (9.96–56.34)	17.90 (10.07–67.02)	16.26 (10.48–50.00)	0.89
fPSA, ng/ml	2.31 (1.15–5.83)	2.38 (1.10–5.66)	2.10 (1.18–6.75)	2.51 (1.21–4.86)	0.96
fPSA/tPSA	0.11 (0.08–0.16)	0.11 (0.08–0.16)	0.11 (0.08–0.16)	0.11 (0.80–0.18)	0.71
PSAD, ng/ml/cm^3^	0.38 (0.19–1.44)	0.38 (0.19–1.48)	0.43 (0.18–1.45)	0.30 (0.20–1.19)	0.90
Prostate volume, ml	43.54 (30.20–63.36)	41.09 (29.47–59.27)	45.64 (29.78–63.86)	46.37 (32.33–73.43)	0.12
Gleason score (GS), %					0.38
Benign	249 (43.9)	164 (46.6)	58 (38.2)	27 (42.9)	
GS ≤ 6	53 (9.3)	32 (9.1)	12 (7.9)	9 (14.3)	
GS = 7	111 (19.6)	64 (18.2)	39 (25.7)	8 (12.7)	
GS = 8	53 (9.3)	33 (9.4)	14 (9.2)	6 (9.5)	
GS = 9	82 (14.5)	49 (13.9)	24 (15.8)	9 (14.3)	
GS = 10	19 (3.4)	10 (2.8)	5 (3.3)	4 (6.3)	
Presence of SVI, %	136 (24.0)	81 (23.0)	39 (25.7)	16 (25.4)	0.79
Presence of LNI, %	138 (24.3)	87 (24.7)	35 (23.0)	16 (25.4)	0.90
Presence of ECE, %	159 (28.0)	97 (27.6)	44 (28.9)	18 (28.6)	0.95

tPSA, prostate-specific antigen; fPSA, free prostate-specific antigen; fPSA/tPSA, the ratio of fPSA to tPSA; PSAD, prostate-specific antigen density; SVI, seminal vesicle invasion; ECE, extracapsular extension; LNI, lymph node invasion.

### Selection of radiomic features

In total, 2,632 radiomic features were extracted from the ROI of T2WI and DWI MRI images. Among them, 1,939 features exhibited strong reproducibility with both inter- and intraobserver correlation coefficients over 0.80. The t-test filtered 1,317 features that were significantly related to the malignancy of prostate masses, of which 238 were retained after Spearman correlation analysis. Finally, the LASSO algorithm with five-fold cross-validation selected 18 radiomic features that are optimal for malignant prostate mass prediction. The features’ selection process using LASSO is demonstrated in **Supplementary Figure S1**. The correlation matrix and clustered heatmaps for the selected features are displayed in **Supplementary Figures S2**, **S3**, respectively.

### Establishment of ML models

**Figure 3:**
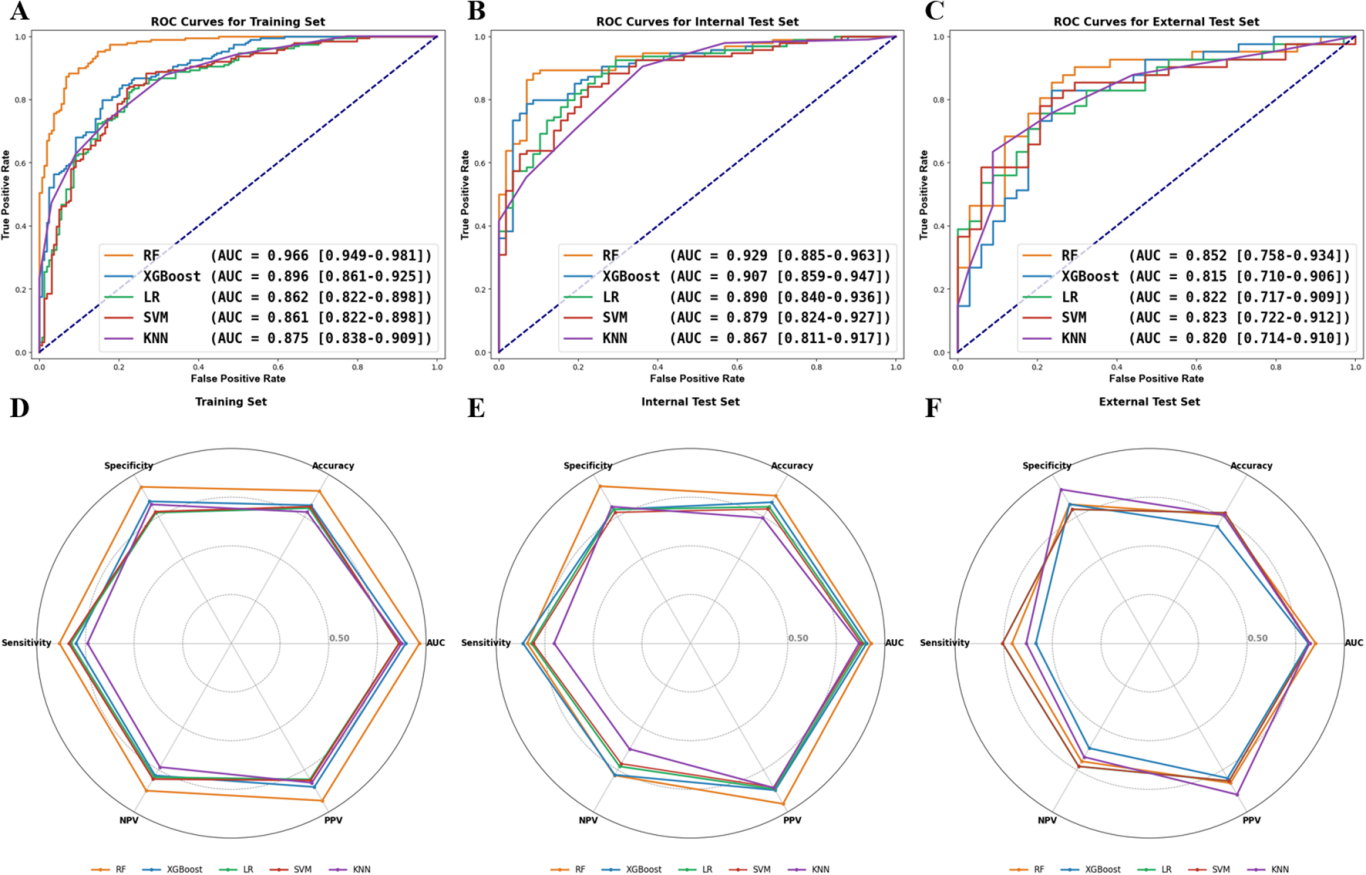
Predictive performances of the five machine learning models. The receiving operating curve (ROC) analysis of the established models in the training **(A)**, internal test **(B)**, and external test **(C)** sets. The models’ predicting metrics radar plot in the training **(D)**, internal test **(E)**, and external test **(F)** sets.

Using the chosen radiomic features and grid search, five ML models were successfully built for differentiating malignant from benign prostate masses in the training set. As shown in **Figures 3A–C**, the RF model obtained the highest AUCs, with 0.966 (95% CI: 0.949–0.981) in the training set, 0.929 (95% CI: 0.885–0.963) in the internal test set, and 0.852 (95% CI: 0.758–0.934) in the external test set. The XGBoost followed, with AUCs of 0.896 (95% CI: 0.861–0.925), 0.907 (95% CI: 0.859–0.947), and 0.815 (95% CI: 0.710–0.906) in the training, internal test, and external test sets, respectively. The LR, SVM, and KNN models ranged as the third, fourth, and fifth predicting models. Meanwhile, the RF model exhibited excellent accuracies, with 0.903, 0.875, and 0.760 across the three datasets (**Figures 3D–F**). The predictive abilities of established ML models in the training, internal test, and external test sets are summarized in **Table 2**.

**Table 2:** The predicting performances of the established five machine learning models in the training, internal test, and the external test sets.

Dataset	Model	AUC (95% CI)	ACC	SPE	SEN	NPV	PPV
Training set	RF	0.966 (0.949–0.981)	0.903	0.927	0.883	0.874	0.933
	XGBoost	0.896 (0.861–0.925)	0.818	0.841	0.798	0.784	0.852
	LR	0.862 (0.822–0.898)	0.801	0.774	0.824	0.794	0.807
	SVM	0.861 (0.822–0.898)	0.810	0.780	0.835	0.805	0.813
	KNN	0.875 (0.838–0.909)	0.778	0.823	0.739	0.734	0.827
Internal test set	RF	0.929 (0.885–0.963)	0.875	0.931	0.840	0.783	0.952
	XGBoost	0.907 (0.859–0.947)	0.836	0.793	0.862	0.780	0.871
	SVM	0.879 (0.824–0.927)	0.796	0.776	0.809	0.714	0.854
	LR	0.890 (0.840–0.936)	0.809	0.793	0.819	0.730	0.865
	KNN	0.867 (0.811–0.917)	0.743	0.810	0.702	0.627	0.857
External test set	RF	0.852 (0.758–0.934)	0.760	0.824	0.707	0.700	0.829
	XGBoost	0.815 (0.710–0.906)	0.693	0.824	0.585	0.622	0.800
	LR	0.822 (0.717–0.909)	0.773	0.794	0.756	0.730	0.816
	SVM	0.823 (0.722–0.912)	0.773	0.794	0.756	0.730	0.816
	KNN	0.820 (0.714–0.910)	0.760	0.912	0.634	0.674	0.897

RF, random forest; XGBoost, eXtreme Gradient Boosting; LR, logistic regression; SVM, support vector machine; KNN, k-nearest neighbor; AUC, area under the ROC curve; CI, confidential interval; ACC, accuracy; SEN, sensitivity; SPE, specificity; PPV, positive predictive value; NPV, negative predictive value.

### Comparison of ML models

**Figure 4:**
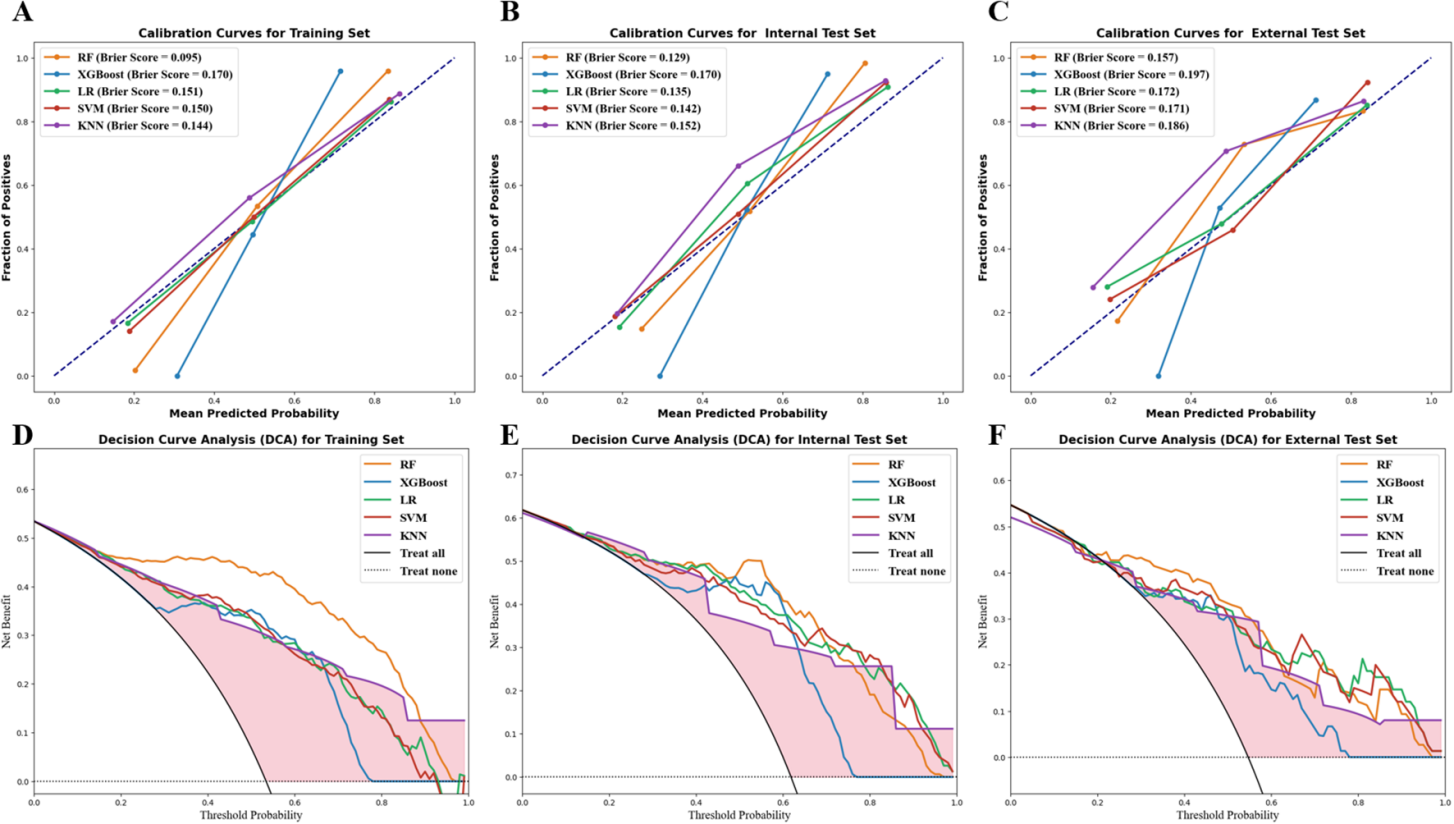
Evaluation of model’s clinical usefulness. Calibration curve analysis of the five models in the training **(A)**, internal test **(B)**, and external test **(C)** sets. Decision curve analysis of the five models in the training **(D)**, internal test **(E)**, and external test **(F)** sets.

**Table 3** lists the DeLong test analysis comparing AUCs of the RF model with other ML models. As a result, except for the external test set, the RF model exceeded the other four models for predicting malignant prostate mass, with all DeLong test *P* values being less than 0.05. Furthermore, the RF model demonstrated optimal calibration across the training, internal test, and external test sets, as indicated by the lowest Brier scores and well-aligned calibration curves (**Figures 4A–C**). Moreover, it achieved the highest net benefit across most threshold probabilities in the decision curve analysis within all three datasets (**Figures 4D–F**). These results affirm the RF model’s predictive reliability and its clinical utility for guiding decision making across diverse datasets.

**Table 3:** Results of DeLong test analysis comparing AUCs of the RF model with other machine learning models.

Dataset	Models	*Z* score	*P* value
Training set	RF vs. XGBoost	7.132	<0.001
	RF vs. LR	7.510	<0.001
	RF vs. SVM	7.155	<0.001
	RF vs. KNN	7.284	<0.001
Internal test set	RF vs. XGBoost	2.118	0.034
	RF vs. LR	2.362	0.018
	RF vs. SVM	2.667	0.008
	RF vs. KNN	2.955	0.003
External test set	RF vs. XGBoost	2.140	0.032
	RF vs. LR	0.957	0.339
	RF vs. SVM	0.794	0.427
	RF vs. KNN	0.935	0.350

AUC, area under the ROC curve**;** RF, random forest; XGBoost, eXtreme Gradient Boosting; LR, logistic regression; SVM, support vector machine; KNN, k-nearest neighbor.

### SHAP interpretation of the RF model

**Figure 5:**
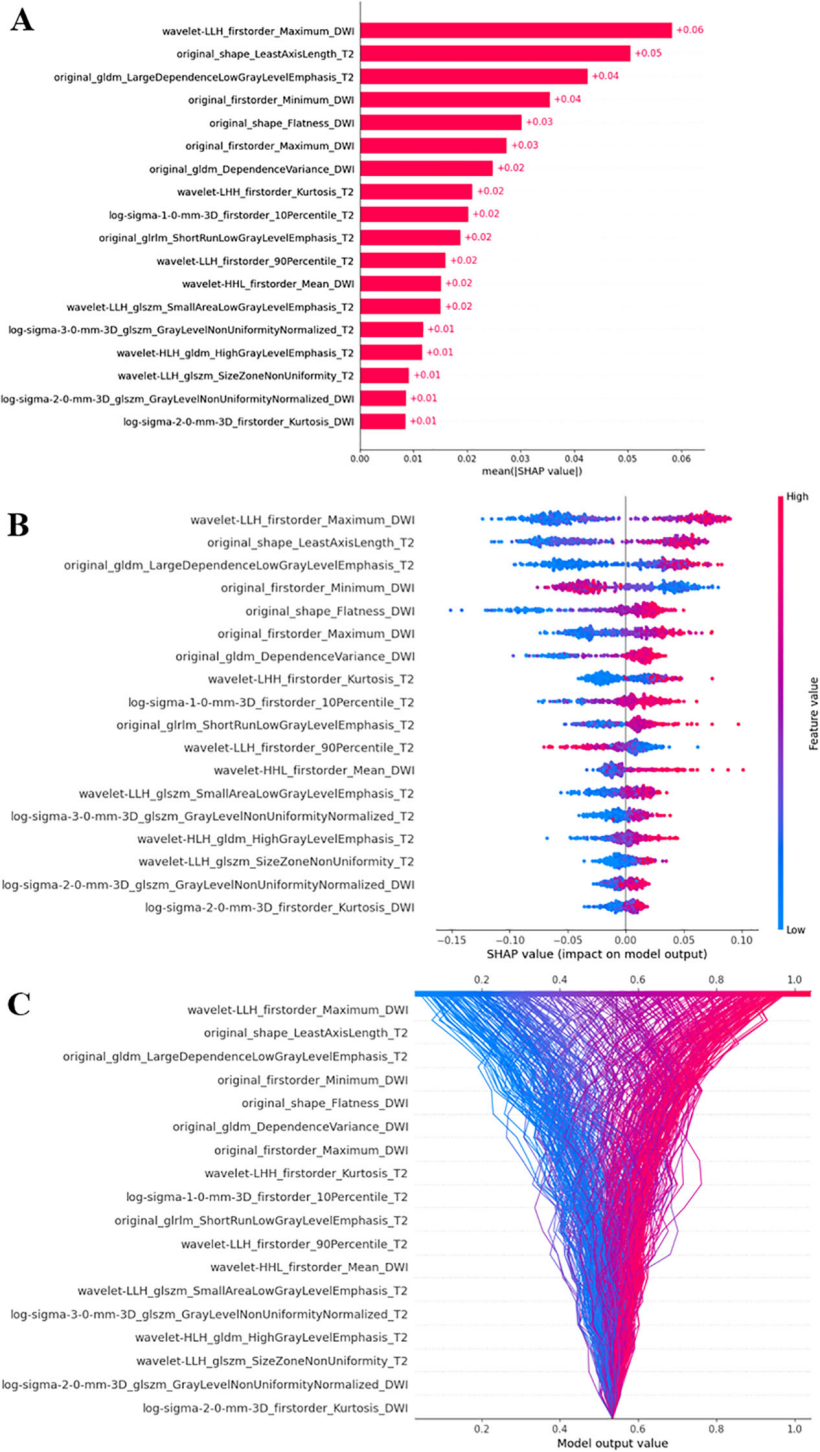
The SHAP analysis of the RF model. **(A)** The SHAP bar plot indicated the contributing values of the radiomic features for RF predictions. **(B)** The SHAP bee-swarm plot demonstrated the positive or negative correlation between radiomic expression and RF output. The x-axis represents the SHAP values, whereas the y-axis lists the radiomic features and their respective values. Each point represents an individual sample, with red points indicating higher feature values and blue points indicating lower values. The spread of points along the x-axis reflects how much each feature influences the model’s prediction, with a wider distribution suggesting that many samples exhibit similar SHAP values for that feature. **(C)** The SHAP decision plot showcased the influences of all contributing features on the final predicting probabilities. The vertical gray line represents the model’s base value. The colored lines show individual predictions, illustrating how each feature either increases or decreases the predicted value relative to the base value. Each feature’s value is indicated next to its respective line. Starting at the bottom, the prediction lines show how SHAP values accumulate to the final model score at the top. Red lines correspond to higher feature values, whereas blue lines correspond to lower feature values. SHAP, SHapley Additive exPlanations; RF, random forest.

**Figure 6:**
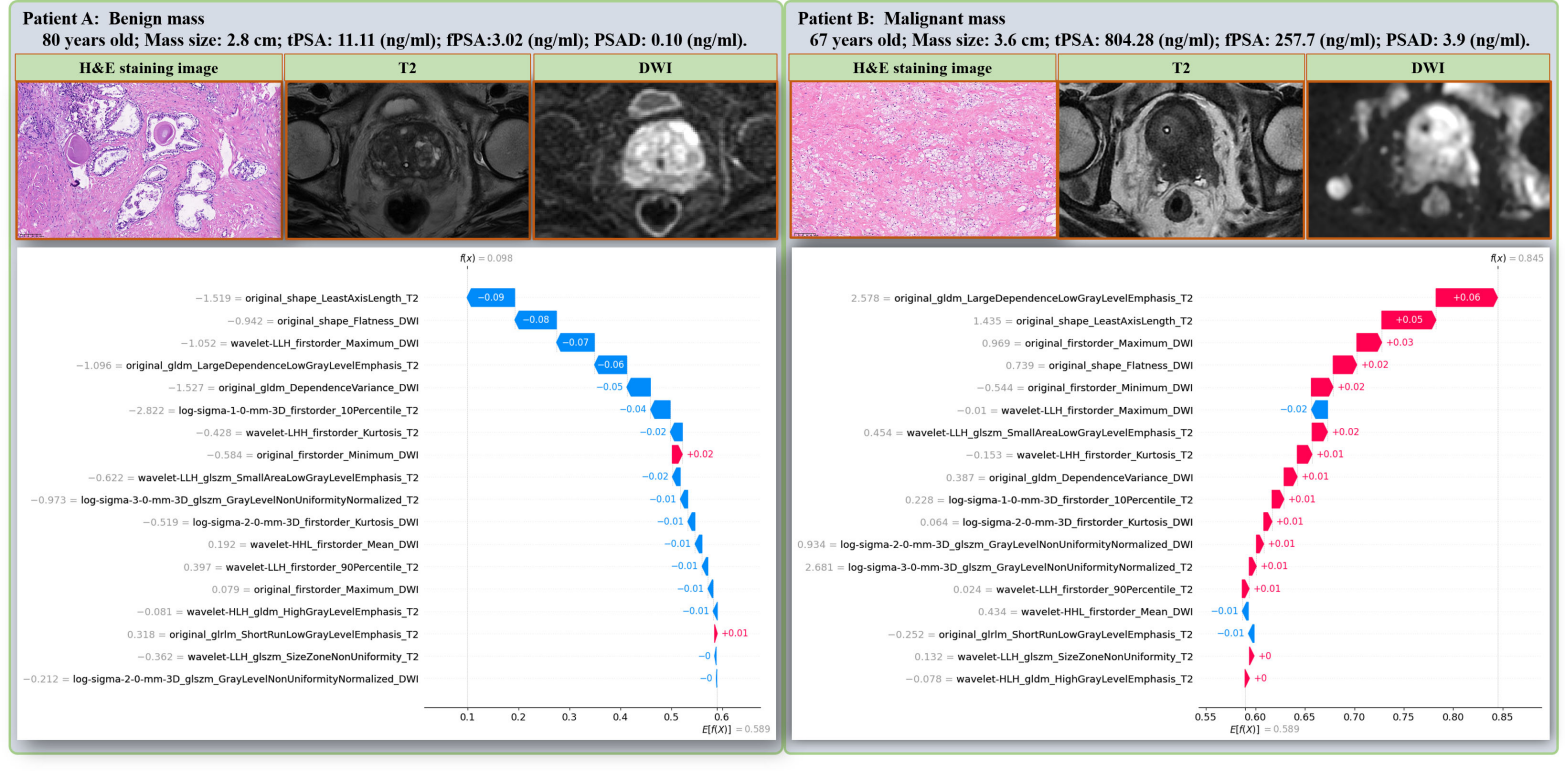
Two representative cases that were successfully differentiated as benign **(A)** or malignant **(B)** prostate mass using the RF model. The distinct contributions of each radiomic features within the RF model for individual predictions are illustrated using the SHAP waterfall plot. RF, random forest; SHAP, SHapley Additive exPlanations.

SHAP was applied to uncover the prediction process of the RF model. As illustrated in **Figure 5A**, the top three contributing radiomic features for malignant prostate mass prediction were wavelet-LLH_firstorder_Maximum_DWI (+0.06), original_shape_LeastAxisLength_T2 (+0.05), and original_gldm_LargeDependenceLowGrayLevelEmphasis_T2 (+0.04). This demonstrates that wavelet-LLH_firstorder_Maximum_DWI was the most influential feature in predicting malignancy, with the model placing the greatest weight on this feature when determining whether a prostate mass is malignant. Following closely in importance were original_shape_LeastAxisLength_T2 and original_gldm_LargeDependenceLowGrayLevelEmphasis_T2, which, although contributing slightly less, still played a significant role in the prediction. Except for the original_firstorder_Minimum_DWI, and the wavelet-LLH_firstorder_90Percentile_T2, all other features were positively correlated with malignancy of prostate mass (**Figure 5B**). This indicates that the majority of the radiomic features in the model were directly related to the likelihood of a prostate mass being malignant. Specifically, as the values of these features increased, the probability of malignancy also increased, emphasizing their importance in differentiating benign from malignant prostate masses. The SHAP decision plot demonstrates the influences of all contributing features on the final predicting probability (**Figure 5C**). In this plot, each point represents an individual prediction, and the position along the x-axis reflects the cumulative contribution of all features to the model’s predicted outcome. Features with higher SHAP values push the prediction toward a higher probability of malignancy, whereas features with lower SHAP values move the prediction toward a lower probability. Moreover, **Figure 6** highlights two representative cases that differentiated benign and malignant prostate mass, illustrating the distinct contributions of each of the 18 selected radiomic features within the RF model. These examples help clarify the specific impact of each feature on the model’s predictive output, enhancing our understanding of the role that these features play in assessing malignancy of prostate mass.

## Discussion

In this study, we successfully developed a noninvasive diagnostic model that combines mpMRI radiomics and machine learning to differentiate between benign and malignant prostate masses. The RF model demonstrated excellent predictive performance across both internal and external validation cohorts. Additionally, the model’s decision-making process was elucidated using the SHAP method, providing valuable insights into its prediction mechanism. These findings highlight the potential of this model to support clinical decision making in prostate cancer diagnosis, offering a reliable and noninvasive tool for preoperative identification of malignant prostate masses.

Radiomics-based ML models have attracted considerable attention in medical imaging, particularly for their potential in differentiating benign from malignant prostate masses in a noninvasive manner. Previously, Li et al. (36) developed six ML models using the mpMRI-derived radiomic features to predict PCa in 238 patients. The RF model was proven to be the best classifier in their study, with an AUC value of 0.931. Castaldo et al. (37) calculated the mpMRI radiomics-based risk score in 189 patients, which successfully differentiated clinically significant PCa from other prostate conditions. Li et al. (38) used mpMRI radiomic features and the LASSO algorithm to develop a diagnostic model for 236 subjects, yielding an AUC value of 0.895–0.956 in differentiating PCa and begin prostate mass. All of their studies confirmed the predictive values of mpMRI radiomic features for malignancy of prostate masses. In consistence with their studies, using a four-step feature selection process, 18 radiomic features were chosen in our study for five ML models’ establishment, which all satisfactorily predicted malignant from benign prostate masses, with AUCs ranging from 0.815 to 0.929 in the test sets. Differing from their findings that based on single center cohorts, our study included external validations, improving the generalization abilities of our models.

More recently, several researches constructed mpMRI radiomic model for PCa diagnosis on the basis of multicenter datasets and ML methods. For example, studies by Mylona et al. (39) demonstrated the effectiveness of mpMRI radiomic features in distinguishing malignant from benign prostate masses. These studies primarily focused on single-modal approaches or combined data from different imaging modalities without explicitly applying feature fusion methods for a more comprehensive exploration of diagnostic data. In contrast, our study adopted a feature-fusion approach, combing the T2WI and DWI MRI-derived radiomic features. By incorporating multimodal radiomic data, we were able to select the most optimal set of features, capturing a broader spectrum of tumor characteristics. This fusion of features provides a more robust and comprehensive representation of prostate mass heterogeneity, which is essential for improving diagnostic accuracy. Besides, the predictive performances of the constructed ML models were compared using the DeLong test, calibration curve, and decision curve analysis, and the most optimal ML model for predicting malignant prostate masses was determined. The RF model outperformed the others, exhibiting the most robust performances in both the training (AUC: 0.966), internal test (AUC: 0.929), and external test (AUC: 0.852) sets, highlighting the superiority of the RF model in predicting malignant prostate masses and underscoring the value of incorporating multimodal radiomic data for improving diagnostic precision.

To be noted, the RF model exhibited weaker statistical significance in the external test set compared to other models in the DeLong test, which may be attributed to the following factors: First, the external dataset was derived from a different institution, and variations in imaging acquisition protocols, scanning parameters, and patient demographics may have impacted the model’s generalizability, leading to a reduced discriminatory ability. Second, the relatively smaller sample size in the external test set may have limited the statistical power of the DeLong test, making it more challenging to detect subtle differences in AUC values. Additionally, although the RF model still achieved the highest AUC, the differences between models were smaller in the external set than in the internal test set, further affecting statistical significance. Future studies should incorporate larger, multicenter datasets to enhance the model’s robustness and generalizability.

The use of the ML model in clinical practice is still met with skepticism, primarily due to the perceived “black box” nature of many algorithms (40, 41). Lack of interpretability remains a barrier, with critics highlighting the need for transparency and reliability in clinical decision-making tools (42, 43). Recent studies have increasingly applied interpretable methods, such as SHapley Additive exPlanations (SHAP), to elucidate the contribution of individual features, thus promoting acceptance of ML-based diagnostic tools in clinical settings (44–46). To the best of our knowledge, there has been no previous study investigating mpMRI radiomics-based interpretable ML model using the SHAP method for predicting malignant prostate masses. Our findings demonstrate that the RF model achieved the best performance, suggesting that RF may offer greater stability and predictive accuracy in multicenter settings. It is therefore chosen to explore the underlying prediction logics by incorporating SHAP. As a result, we identified specific radiomic features, such as wavelet-LLH_firstorder_Maximum_DWI and original_shape_LeastAxisLength_T2, that contribute significantly to malignancy predictions. The contributed relations of the 18 selected radiomic features were successfully illustrated using the SHAP bar plot, SHAP bee-swarm plot, and SHAP decision plot. This approach not only enhances model transparency but also allows clinicians to understand the influence of individual features on diagnostic predictions. In addition, the precise prediction of RF model based on the selected radiomic features may be due to the underlying correlations between MRI radiomics and tumor biological heterogeneity. For example: the wavelet-LLH_firstorder_Maximum_DWI suggests the presence of highly variable cellular structures, which can be indicative of tumor aggressiveness and heterogeneity, often linked to increased cellular density and irregularity. The original_shape_LeastAxisLength_T2 may be associated with the tumor’s morphological characteristics, such as its invasive potential or spatial expansion patterns, which can reflect aggressive tumor growth. Lastly, the original_gldm_LargeDependenceLowGrayLevelEmphasis feature, extracted from T2-weighted images, is sensitive to areas with low gray-level variation, often correlating with stromal changes and microvascular structures in the tumor microenvironment.

Several limitations of this study should be acknowledged. First, the retrospective nature of this study results in an inevitable selection bias, which may affect the representativeness of the study population and the generalizability of the findings. This underscores the need for prospective studies with predefined inclusion criteria and systematic follow-up protocols, as well as external validation in larger, independent cohorts, to confirm the robustness and clinical applicability of our results. Second, although the use of multicenter datasets increases generalizability, the sample size still does not adequately reflect the broader diversity of prostate cancer patients. A large-scale international multicenter study design is expected in future researches. Third, although SHAP improved model interpretability by identifying influential features, it does not entirely resolve the challenges that clinicians face in applying machine learning outputs in clinical settings, as the underlying molecular explanations of radiomics-based model remain unrevealed. Last but not least, the manual delineation of prostate mass region not only is time- and labor-dependent but also faces the reproducibility issue. Auto or semiauto MRI segmentation tools for prostate mass are urgently needed.

In conclusion, this study demonstrates the potential usage of the mpMRI radiomics-based interpretable machine learning model for differentiating malignant from benign prostate masses. The successful application of the SHAP method provides further transparency in model predictions, a critical step toward clinical adoption. This approach holds promise for improving preoperative prostate cancer diagnosis and guiding personalized treatment strategies.

## Data Availability

The original contributions presented in the study are included in the article/**Supplementary Material**. Further inquiries can be directed to the corresponding authors.
