# Supplementary material for: Interpretable multiparametric MRI radiomics-based machine learning model for preoperative differentiation between benign and malignant prostate masses: a diagnostic, multicenter study
Source: Front Oncol. 2025 May 5;15:1541618. doi: 10.3389/fonc.2025.1541618 (PMC12086068; doi:10.3389/fonc.2025.1541618)

**Supplementary S1** The detailed MRI parameters in this study

At Center 1 (using the GE Discovery MR750W, General Healthcare, Milwaukee, USA): the optimized T2WI parameters included a repetition time (TR) of 3000 ms, echo time (TE) of 95 ms, field of view (FOV) of 200 × 200 mm², matrix size of 240 × 240, slice thickness of 3 mm, with no interslice gap. DWI was acquired using an echo planar imaging (EPI) sequence with TR of 6000 ms, TE of 80 ms, FOV of 220 x 220 mm², matrix size of 128 x 128, slice thickness of 4 mm, no interslice gap, and b-values of 0, 800, and 1200 s/mm². ADC maps were automatically generated to support the assessment of lesion cellularity and tumor characteristics.

At Center 2 (using the Philips Intera Achieva, Best, Netherlands): the optimized T2WI parameters included a TR of 3000 ms, TE of 100 ms, FOV of 220 × 220 mm², matrix size of 276 × 278, slice thickness of 3 mm, with no interslice gap. DWI was acquired using an EPI sequence with TR of 6000 ms, TE of 77 ms, FOV of 260 x 260 mm², matrix size of 104 x 125, slice thickness of 3 mm, no interslice gap, and b-values of 0, 100, 1000, and 2000 s/mm². ADC maps were automatically generated to support the assessment of lesion cellularity and tumor characteristics.

**Fig. S1** The features’ selection process using the LASSO. **(A)** The LASSO logistic regression selected 18 radiomics features at a tuning parameter (λ) of 0.0492388. **(B)** The coefficient profile was plot according to the selected log λ value. **(C)** The selected features and their corresponding weighted values.


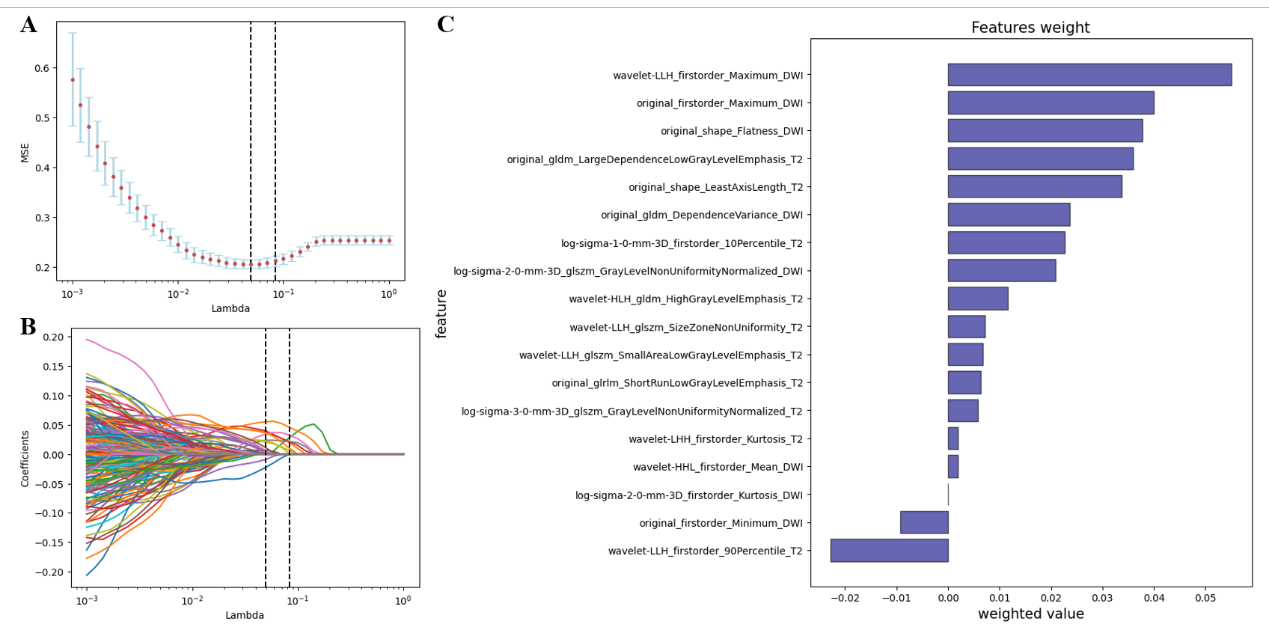


**Fig. S2** The correlation matrix of the selected radiomics features


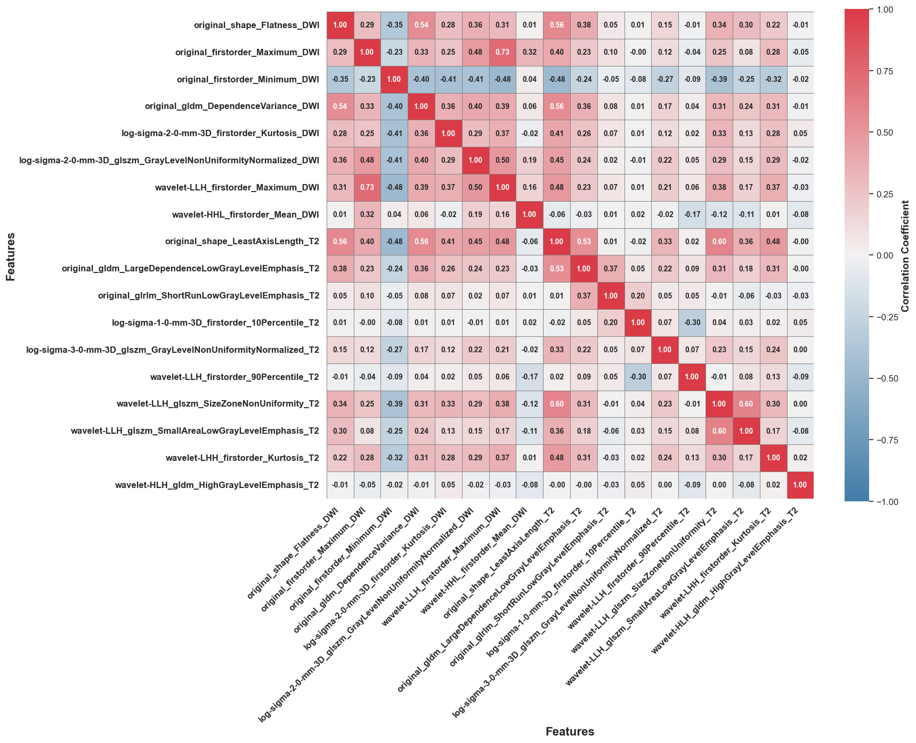


**Fig. S3** The clustered heatmaps of the selected radiomics features


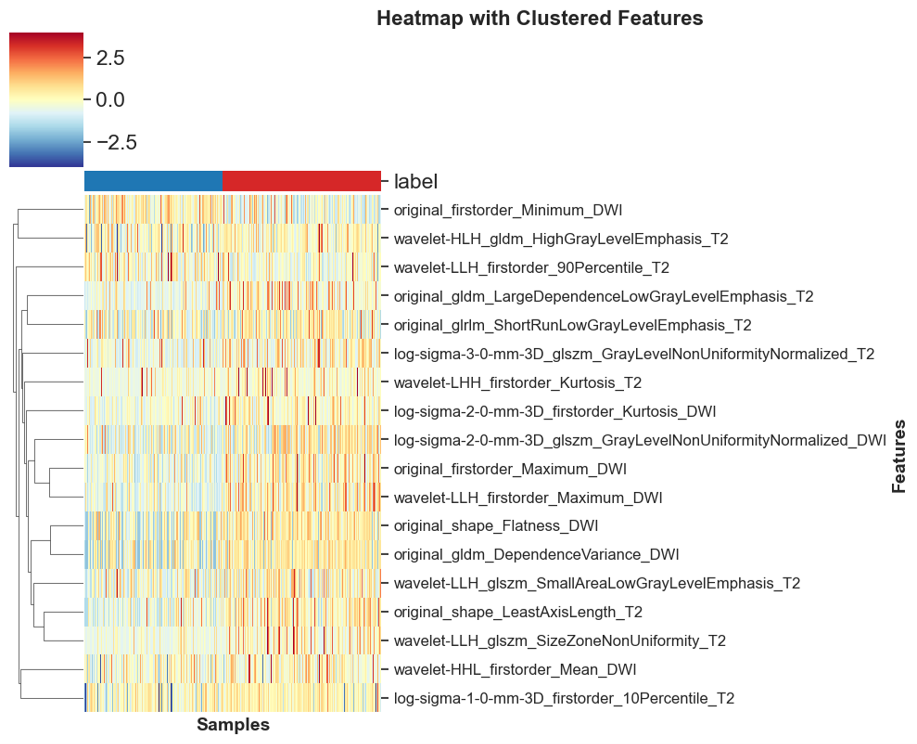

Supplement: Supplementary file 1 [file DataSheet1.docx]
